# Clinical Predictors of Dosimetric Precedence for Deep Inspiratory Breath Hold Radiation Therapy for Breast Cancer

**DOI:** 10.3390/diagnostics16081236

**Published:** 2026-04-21

**Authors:** Alzahra’a Al Matairi, Abdulla Alzibdeh, Issa Mohamad, Ramiz Abu-Hijlih, Wafa Asha, Fadwa Abdel Rahman, Haitham Kanaan, Soha Ahmad, Hikmat Abdel-Razeq, Fawzi Abuhijla

**Affiliations:** 1 Faculty of Medicine, The University of Jordan, Amman 11942, Jordan; 2 Department of Radiation Oncology, King Hussein Cancer Center, Amman 11941, Jordan; 3 Department of Clinical Oncology and Nuclear Medicine, Faculty of Medicine, Suez University, Suez 43518, Egypt; 4 Department of Medical Oncology, King Hussein Cancer Center, Amman 11941, Jordan

**Keywords:** free breathing, deep inspiration breath-hold, radiotherapy, dosimetric predictors

## Abstract

**Background/Objectives**: Radiation therapy for left-sided breast cancer exposes the heart and lungs to incidental radiation, increasing long-term risk of cardiopulmonary morbidity. This study evaluated the dosimetric impact of DIBH compared with free breathing (FB) and explored patient- and treatment-related predictors of benefit. **Methods**: We retrospectively analyzed 90 patients with left-sided breast cancer or chest wall irradiation planned under both FB and DIBH. Dosimetric parameters included mean heart dose (MHD), mean lung dose (MLD), heart V5, and lung V20. Univariable analyses assessed associations between dose reductions (Δ%) and clinical factors (age, BMI, fractionation, boost, nodal fields, and smoking). Multivariable regression identified independent predictors. **Results**: DIBH significantly reduced mean doses to the heart (FB 4.25 ± 1.47 Gy vs. DIBH 2.64 ± 1.28 Gy; Δ −1.60 Gy, *p* < 0.001, and Cohen’s d = 2.46) and lung (FB 14.00 ± 3.45 Gy vs. DIBH 11.64 ± 3.32 Gy; Δ −2.36 Gy, *p* < 0.001, and Cohen’s d = 2.73). Similarly, heart V5 (median Δ −10.5%, *p* < 0.001) and lung V20 (median Δ −5.5%, *p* < 0.001) were significantly improved. Higher BMI was independently associated with smaller relative reductions in MHD (−1.4% per unit, *p* < 0.001) and MLD (−0.31% per unit, *p* = 0.019). Hypofractionation was linked to greater MHD and MLD reductions (+8.9%, *p* = 0.007), (3.7%, *p* = 0.035), respectively. Supraclavicular field irradiation increased lung exposure, while internal mammary chain fields elevated both lung and heart doses. Age showed no significant influence. **Conclusions**: DIBH provides robust and consistent reductions in cardiac and pulmonary radiation doses during left-sided breast irradiation. The degree of sparing is modulated by BMI, fractionation, and nodal field inclusion.

## 1. Introduction

Breast cancer is the most prevalent non-skin cancer in women [1]. Although it has the highest mortality rates, it also has a high chance of cure, especially in the early stages. Radiation therapy (RT) is beneficial for both breast-conserving therapy patients and patients who have undergone radical mastectomies and possess risk factors for relapse. RT is considered a cornerstone of breast cancer treatment. It has been shown to significantly reduce the risk of recurrence and improve survival rates [2,3]. While RT provides valuable advantages in treating breast cancer, it is important to recognize the potential for long-term cardiovascular and pulmonary risks. Studies suggest that these risks, such as cardiovascular mortality and ischemic heart disease (IHD), may influence the efficacy and safety of this therapy, with a potential increase of 7.4% in ischemic coronary heart disease risk per Gray in the mean heart dose (MHD) [4].

Deep inspiration breath hold (DIBH) is a respiratory technique in which patients take a deep breath and hold it during the delivery of radiation. It is mainly used to decrease the potential for developing long-term cardiac and pulmonary toxicity in women receiving radiation therapy for left breast cancer. This method ultimately minimizes the radiation dose to the heart and lung during left breast irradiation, both with and without regional nodal irradiation (RNI). Furthermore, DIBH brings prominent lung-sparing benefits [5]. However, the efficacy of this method may be affected by various clinical factors [6]. An increase in body mass index (BMI) has been associated with alterations in respiratory mechanics and reduced lung function, reflecting both the mechanical and inflammatory effects of obesity [7]. These changes may influence the ability to achieve adequate and consistent lung expansion during DIBH. However, BMI itself is not a direct determinant of DIBH benefit; rather, it likely acts as an indirect predictor through its relationship with inspiratory lung volume. Moreover, inspiratory lung volume has been shown to be a dominant predictor of cardiac dose reduction [8]. Additionally, patients receiving RNI, including internal mammary chain nodes, experience comparable or even greater reductions in the mean heart and left anterior descending artery dose than those receiving breast-only irradiation, highlighting the particular benefit of DIBH in the RNI setting [9].

Despite the well-established benefits of deep inspiration breath hold in reducing cardiac and pulmonary radiation exposure, the magnitude of these benefits can vary depending on patient- and treatment-related factors, such as respiratory capacity, body composition, and the extent of the radiation field. The impact of these clinical variables on dose reduction has not been systematically evaluated. This study therefore aims to quantify the cardiac and pulmonary dose reductions achieved with DIBH in patients undergoing left-sided breast irradiation and to identify the clinical factors that predict maximal dosimetric benefit.

## 2. Methods and Materials

### 2.1. Patient Population and Study Design

Free breathing and DIBH computed tomography (CT) simulations without contrast were obtained from ninety patients originally receiving intact left breast or left chest wall irradiation between January 2019 and December 2021. Patients were included consecutively during the study period. Inclusion criteria were histologically confirmed breast cancer, indication for adjuvant breast/chest wall radiotherapy (±nodal irradiation), and availability of both FB and DIBH CT simulations. Patients with incomplete or non-evaluable datasets were excluded. Patients in this cohort received a variety of standard neoadjuvant and adjuvant chemotherapy regimens, including anthracycline- and/or taxane-based protocols, administered according to tumor biology and institutional guidelines. Data on patient characteristics such as age, body mass index, and smoking status were collected. Multiple heart and lung doses were recorded from both the FB and DIBH plans. This was done to facilitate the analysis of various dose level reductions between the FB and DIBH plans, concerning each patient and technique-specific factors collected.

All patients’ target and organ-at-risk volumes were contoured on both FB and DIBH, then planned for radiotherapy to the whole left breast or whole left breast plus RNI using the DIBH technique. RT was delivered as indicated to the primary site with or without regional nodal irradiation using conventional tangential fields or VMAT techniques. RT dose regimen included conventional and hypofractionation. Conventional fractionation was defined as 50 Gy in 25 fractions (2 Gy per fraction), while hypofractionation was defined as 40.05 Gy in 15 fractions (2.67 Gy per fraction), in accordance with our institutional protocol. All treatment plans, including conventional tangential and VMAT techniques, were generated using the Pinnacle^3^ treatment planning system (version 16.2, Philips Radiation Oncology Systems, Bothell, WA, USA). Dose calculations were performed using a collapsed cone convolution/superposition algorithm with a calculation grid size of 2.5 mm. Treatments were delivered using megavoltage photon beams (primarily 6 MV, with higher energies used as clinically indicated). Both plan types were optimized using identical target coverage objectives (≥95% of the PTV receiving ≥95% of the prescribed dose), while organ-at-risk (OAR) constraints were applied according to the fractionation regimen. For conventional fractionation (50 Gy/25 fx), heart mean dose was constrained to <4 Gy and ipsilateral lung to V20Gy < 20% and V10Gy < 30%; for hypofractionation (40.05 Gy/15 fx), constraints were adjusted for higher dose per fraction, with heart mean dose < 3.5 Gy and ipsilateral lung V17Gy < 20% and V10Gy < 30%. Contralateral lungs and breasts were minimized as low as reasonably achievable. For this investigation, no additional imaging was needed. This retrospective study was approved by the Institutional Review Board. Patients were scanned in a supine position with arms raised above their heads using a breast board fixating tool. Patients did not change their position between FB and DIBH scans. Patients received both vocal guidance and visual feedback from radiation therapists during the DIBH scan. For the DIBH technique, patients used the active breath control (ABC) device with a threshold of ≥1.5 L of inspiratory volume. The apparatus consists of a mouthpiece that is fastened to a spirometer; the patient’s nose is pinched to guarantee that they are breathing only via the device. The radiation therapists can see the patient’s inspiration level since the spirometer is linked to a computer. Pinch valves in the spirometer remotely shut when the patient reaches the necessary threshold, stopping them from breathing in or out beyond it [10].

### 2.2. Target Volume and Organ at Risk Delineation

According to the Radiation Therapy Oncology Group (RTOG) contouring atlas [11], axillary lymph node contouring of levels I, II, and III was based on well-defined anatomic landmarks to ensure consistent and reproducible delineation. Level I is bordered laterally by the medial edge of the latissimus dorsi muscle and extends medially to the lateral border of the pectoralis minor, with anterior and posterior limits defined by the anterior surface of the pectoralis major and the ribs/intercostal muscles, respectively. Level II lies beneath the pectoralis minor, extending from its lateral to medial borders, and is bound anteriorly by the ribs and intercostal muscles. Level III is positioned medial to the pectoralis minor, bound by the anterior surface of the subscapularis muscle posteriorly and the medial border of the pectoralis minor laterally, and extends cranially to the apex near the subclavian vessels. For level IV (supraclavicular) lymph nodes, contours were not mandatory, as the supraclavicular (SCF) field was used for patients who had their treatment plan done with 3DCRT. For contoured cases, the volume extends cranially up to the caudal border of the cricoid cartilage. Caudally, it is delineated at or just above the clavicle. Medially, the volume includes the jugular vein. Laterally, it is bound by the lateral edge of the sternocleidomastoid muscle or, in some cases, the anterior scalene muscle, depending on the anatomic. Anteriorly, the boundary is formed by the sternocleidomastoid muscle or soft tissues overlying it, while posteriorly, the limit can be the anterior surface of the scalene muscles or prevertebral fascia.

For chest wall and residual breast contouring, the ESTRO consensus guidelines were utilized [12]. For post-lumpectomy cases, the residual breast contour extends cranially to just below the inferior edge of the clavicle and caudally to 1–2 cm below the inframammary fold, with medial coverage near the lateral border of the sternum and lateral coverage out to the mid-axillary line (or the visible breast tissue on CT). Anteriorly, the target encompasses the skin surface minus 5 mm, while posteriorly, it is bound by the pectoralis major muscle and the underlying ribs/intercostal muscles. For chest wall contouring of post-mastectomy cases, the cranial border is often placed at or just below the clavicle (covering the second rib area), extending inferiorly to approximately 2 cm below the inframammary fold or to the lowest extent of the chest wall as clinically indicated. Medially, it extends to the lateral sternal border, and laterally, it reaches the mid-axillary line. Anteriorly, the skin surface is included if clinically indicated, and posteriorly, the volume usually encompasses the anterior surface of the ribs or intercostal muscles, with full inclusion of the intercostal muscles if clinically indicated.

### 2.3. Statistical Analysis

#### 2.3.1. Descriptive Analysis

All statistical analyses were performed using IBM SPSS Statistics version 27 (IBM Corp., Armonk, NY, USA) and Microsoft Excel 2019. A two-sided *p*-value < 0.05 was considered statistically significant. Normality was assessed using the Shapiro–Wilk Test, and variables were summarized as mean ± standard deviation (SD) if normally distributed or median with interquartile range (IQR) if not normal. Categorical variables were presented as frequencies and percentages.

#### 2.3.2. Comparison of Dosimetric Parameters

The primary objective was to compare heart and lung doses between FB and DIBH plans. Paired t-tests were used for normally distributed differences, while the Wilcoxon signed-rank test was applied for non-normal data. Heart V5 was defined as the percentage volume of the heart receiving ≥ 5 Gy, and Lung V20 was defined as the percentage volume of the ipsilateral lung receiving ≥ 20 Gy. Relative reductions (ΔMHD, ΔMLD, ΔLung V20, and ΔHeart V5) were calculated as:

Δ = (FB − DIBH)/FB × 100

Effect sizes were reported using Cohen’s d for paired data. Results were expressed as mean or median reductions with 95% confidence intervals (CIs). Boxplots were used to visualize differences, and individual patient reductions were presented using a waterfall plot.

#### 2.3.3. Univariable Analysis of Predictors of Dosimetric Benefit

Predictors of (ΔMHD, ΔMLD, ΔLung V20, and ΔHeart V5) were explored using appropriate statistical tests according to variable type and distribution. Pearson correlation was used for normally distributed continuous variables and Spearman correlation for skewed data. Correlation coefficients (r) and *p*-values were reported. For binary predictors, independent t-tests were applied for normally distributed variables; Mann–Whitney U tests were used otherwise.

#### 2.3.4. Multivariable Analysis

Variables with *p* < 0.10 in univariable analyses, along with clinically relevant covariates, were entered into multivariable linear regression models to identify independent predictors of percentage dosimetric reductions. Model assumptions were evaluated by inspection of residual plots. Normality of residuals, homoscedasticity, and absence of influential outliers were confirmed. Multicollinearity was assessed using variance inflation factors (VIFs). Regression coefficients (β), 95% confidence intervals (CIs), and *p*-values were reported.

## 3. Results

### 3.1. Baseline Characteristics

The baseline characteristics of the study cohort are summarized in Table 1. Among the 90 patients included, the majority were non-smokers (73.3%), and 64.4% had a mastectomy. Conventional dose regimens were more common (70.0%) than hypofractionated regimens (30.0%). Most patients received supraclavicular fossa irradiation (81.1%), and tangent radiation was the predominant technique (90.0%). The median age was 44.0 years (IQR 36.0–46.0), and the mean BMI was 29.52 ± 5.15 kg/m^2^.

### 3.2. Dosimetric Comparison Between FB and DIBH

**Figure 1:**
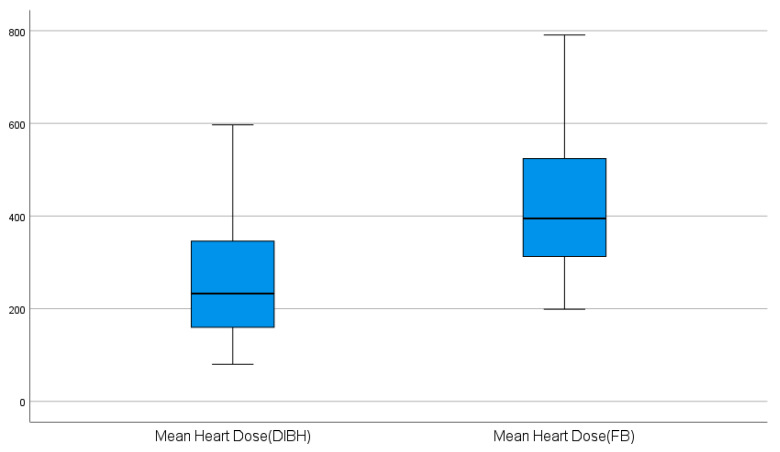
Boxplots of mean heart dose in free breathing vs. DIBH.

**Figure 2:**
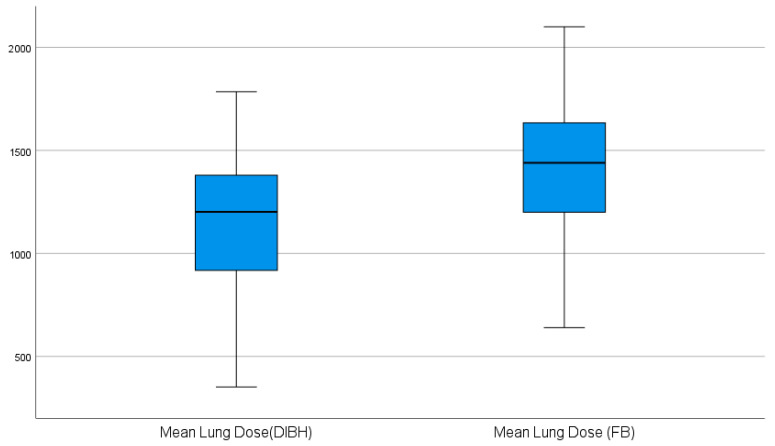
Boxplots of mean lung dose in free breathing vs. DIBH.

The dosimetric outcomes for the lung and heart under FB and DIBH are summarized in Table 2 and Table 3. For normally distributed parameters, DIBH significantly reduced both mean lung and mean heart doses compared with FB (Table 2, Figure 1 and Figure 2). Similarly, for non-normally distributed parameters, DIBH significantly lowered heart V5 and lung V20 doses (Table 3). These results indicate that DIBH consistently decreases radiation exposure to both the heart and lungs across all evaluated parameters.

### 3.3. Univariable Analysis of Factors Associated with Dosimetric Changes

Univariable analyses were performed to evaluate associations between patient- and treatment-related factors and relative dosimetric reductions achieved with DIBH (Table 4). A higher BMI was significantly associated with lower relative dose reductions in Δ%MHD, Δ%MLD, and Δ%Lung V20. The tumor bed threshold demonstrated a modest positive correlation with Δ%MHD, Δ%Lung V20, and Δ%Heart V5, with a borderline association for Δ%MLD. Age was not significantly correlated with any of the dosimetric reductions.

Table 5 summarizes univariable associations between binary patient- and treatment-related factors and relative dosimetric reductions achieved with DIBH. Patients treated with hypofractionated regimens demonstrated significantly larger reductions in mean heart dose (Δ%MHD: −9.1%, 95% CI −15.6 to −2.7; *p* = 0.006) and mean lung dose compared with conventional fractionation. Reductions in lung V20 and heart V5 were also significantly larger in the hypofractionated group.

Regarding regional nodal irradiation, inclusion of the supraclavicular fossa was associated with significantly smaller reductions in mean lung dose and lung V20, while changes in mean heart dose and heart V5 were not significant. Axillary irradiation was not significantly associated with differences in dosimetric reductions. In contrast, internal mammary chain irradiation was associated with smaller reductions in mean lung dose and heart V5, with nonsignificant trends toward smaller reductions in mean heart dose and lung V20.

### 3.4. Multivariable Analysis of Factors Associated with Dosimetric Changes

Table 6 summarizes the multivariable analysis of factors associated with relative dosimetric reductions. Collinearity among predictors was assessed using variance inflation factors (VIFs), all of which were <3, indicating no significant multicollinearity. Higher BMI was independently associated with smaller reductions in mean heart dose (Δ%MHD: −1.4% per unit increase; *p* < 0.001) and mean lung dose (Δ%MLD: −0.31% per unit; *p* = 0.019).

To provide transparency regarding potential confounding between fractionation and radiation technique, the cross-distribution of fractionation by technique was examined: among conventional fractionation patients, 56 (88.9%) received tangential fields and seven (11.1%) received VMAT; among hypofractionated patients, 25 (92.6%) received tangential fields, and two (7.4%) received VMAT. Thus, the majority of patients in both fractionation groups were treated with tangential fields. Given the very small number of patients treated with VMAT (*n* = 9, 10% overall), this variable was not incorporated into the multivariable model, as reliable estimates cannot be obtained with such a limited sample size. While residual confounding by radiation technique cannot be entirely ruled out, the predominance of tangential field use in both fractionation groups (89% conventional, 93% hypofractionated) suggests that any such effect is likely minimal.

Compared with conventional treatment, hypofractionated treatment was independently associated with greater reductions in mean heart dose, mean lung dose, and heart V5.

Inclusion of supraclavicular fossa irradiation was independently associated with lower reductions in mean lung dose (Δ%MLD: −4.7%; *p* = 0.017) and lung V20 dose (Δ%Lung V20: −8.8%; *p* = 0.005). Neither age nor internal mammary chain irradiation was significantly associated with dosimetric outcomes.

Additional subgroup analyses according to smoking status, including comparisons of ΔMHD, ΔLung_Mean, ΔLung_V20, and ΔHeart_V5, are provided in Supplementary Tables S1 and S2.

## 4. Discussion

This retrospective study analyzed 90 patients with left-sided breast cancer who underwent radiation therapy, aiming to assess the protective effect of the DIBH technique in reducing lung and heart radiation exposure and to investigate the clinical predictors of its dosimetric benefit.

Our data demonstrate that implementing DIBH during left-sided breast radiotherapy results in substantial dosimetric benefits. Across all endpoints, mean cardiac and pulmonary doses were significantly lower with DIBH than with free breathing. For example, the mean heart dose fell from ~4.25 Gy (424.7 cGy) on average to ~2.64 Gy (264.3 cGy), a reduction of about 38%, and the mean ipsilateral lung dose decreased by ~17% (from 14.00 Gy to 11.64 Gy). These reductions were highly significant (*p *< 0.001) with very large effect sizes (Cohen’s d > 2). Low-dose metrics likewise improved: heart V5 and lung V20 were markedly lower in DIBH (Table 3). These findings are consistent with previous reports demonstrating the efficacy of DIBH in sparing organs at risk during breast cancer radiotherapy [9,13].

In our univariable and multivariable analyses, patient and treatment factors influenced the degree of cardiac and pulmonary sparing. Notably, a higher BMI correlated with a smaller relative dose reduction, meaning obese patients benefited less from DIBH. A possible explanation is that heavier patients achieve less lung inflation or have larger baseline heart–chest wall distances, thereby reducing displacement effects [7]. We also found that hypofractionation was associated with greater percentage reductions in the mean heart dose and heart V5 than conventional fractionation. While the total dose and per-fraction dose differ between these regimens, our finding suggests DIBH provides relatively more cardiac sparing under hypofractionated schedules. Regarding pulmonary metrics, hypofractionation was also associated with greater reductions in the mean lung dose, while the association with lung V20 showed a similar trend but did not reach statistical significance in multivariable analysis.

Regarding nodal fields, SCF irradiation was associated with smaller dosimetric reductions in lung metrics in multivariable analysis, indicating that DIBH offers relatively less benefit when SCF fields are included. IMC irradiation was not significantly associated with changes in either the heart or lung dose, though point estimates suggested slightly smaller reductions.

Age had no significant impact on dosimetric gains, indicating that the mechanical benefit of DIBH is independent of this patient factor.

Our results align strongly with the fact that DIBH substantially reduces cardiac exposure in left-sided breast radiotherapy. For instance, Falco et al. reported mean heart dose reductions from 3.48 Gy (FB) to 2.10 Gy (DIBH) in a large cohort [14], and Knöchelmann et al. reported a comparable reduction from 2.64 Gy to 1.39 Gy (*p *< 0.001) [15]. These correspond to ~36–47% relative decreases, comparable to our observed ~38% reduction. Likewise, a recent analysis reported that DIBH lowered the mean heart dose by a median of 43.6% across patients [16]. Our findings also agree with those reports of improved low-dose heart metrics, such as V5. These consistent patterns corroborate that DIBH is highly effective for cardiac sparing, as emphasized in the literature [17].

Pulmonary dosimetry under DIBH was also favorably altered. In our cohort, the ipsilateral lung V20 and mean lung dose decreased significantly with DIBH. This matches prior work: Oechsner et al. found average reductions of ~19% in the mean lung dose and ~24% in V20 [18]. Similarly, Zurl et al. reported that the mean dose to the ipsilateral lung was significantly reduced by 15% with DIBH [19]. In contrast, Hayden et al. observed no significant change in lung V20 or mean dose [20]; such discrepancies may reflect differences in patient anatomy or target volume, as their study included internal mammary irradiation with tangential fields. Overall, our data and the literature suggest that lung sparing from DIBH is generally achievable but may vary between populations and planning techniques.

The negative correlation we found between BMI and dose reduction is supported by multiple other studies, which likewise reported that a higher BMI was associated with less relative heart-sparing benefit [8,21,22,23]. They recommend that DIBH be prioritized in lower-BMI patients, consistent with our finding that leaner women had larger percentage dose reductions. With respect to nodal irradiation, our results agree with the known effects of IMC fields. Nguyen et al. showed that DIBH still spares the heart even when internal mammary nodes are treated, but the inclusion of IMC raises the absolute heart dose and estimated risk significantly [24]. Our findings indicate that hypofractionation enhances the overall dosimetric benefits of DIBH, improving the sparing of both cardiac and pulmonary structures. These findings align with planning studies, such as Burchardt et al., which reported that DIBH combined with tangential hypofractionated techniques (tVMAT or 3D-FiF) achieved lower mean heart and LAD doses while also controlling lung exposure [25]. Together, these results suggest that hypofractionated DIBH protocols provide robust, broad organ-at-risk protection, supporting their use in left-sided breast radiotherapy. Finally, the clinical relevance of heart dose reduction is underscored by Darby et al., who quantified a 7.4% increase in major coronary events per 1 Gy mean heart dose [4]. In our study, the mean heart dose dropped by ~1.6 Gy (160 cGy), implying a substantial potential decrease in long-term cardiac risk.

Our study benefits from a paired-plan design, comparing FB and DIBH plans within the same patients, which controls for inter-patient variability. We used consistent planning techniques and evaluated multiple dose metrics (mean dose and Vx values) for both heart and lung, with rigorous statistical testing and effect size reporting. The multivariable analyses accounted for potential confounders (no collinearity detected, VIF < 3), strengthening causal interpretation. However, limitations include its retrospective, single-institution nature and relatively small sample (*N* = 90), which may limit generalizability. The majority of patients received tangential fields, with only 10% planned with VMAT, so our findings may not extend to all planning techniques. Importantly, we report only dosimetric endpoints; no clinical toxicity or outcome data are available, and long-term benefits are inferred from dose reductions only. In addition, comparisons between conventional and hypofractionated regimens were based on physical dose reductions without biologically equivalent dose (EQD2) normalization; therefore, differences in fraction size and total dose to organs at risk are not accounted for, which may limit the biological interpretability of these comparisons.

We also did not analyze substructures (e.g., LAD) or other OARs such as the contralateral breast. Additionally, only patients who were able to successfully perform the DIBH technique were included in the analysis; therefore, our results may not reflect outcomes in patients who are unable to perform DIBH, and future studies should investigate strategies to improve the feasibility of this technique across broader patient populations. Finally, patient selection bias is possible (only those able to perform DIBH were included), and the fractionation groups were uneven (70% conventional), which could influence the observed differences between regimens.

## 5. Conclusions

In summary, our results demonstrate that DIBH provides significant cardiac and pulmonary dose sparing compared with free breathing in left-sided breast/chest wall radiotherapy. DIBH offers the greatest benefit in lower-BMI patients and those undergoing hypofractionation. Given these advantages, the incorporation of DIBH should be strongly considered in left-sided treatments. Future work should continue to refine patient selection, streamline implementation, and ultimately assess whether the dosimetric improvements translate into reduced cardiac and pulmonary morbidity.

## Figures and Tables

**Table 1:** Baseline characteristics of the study cohort (*N* = 90).

Characteristics	Value
Smoking status, *n* (%)	
Non-smoker	66 (73.3)
Smoker	18 (20.0)
Ex-smoker	6 (6.7)
Surgical procedure, *n* (%)	
Mastectomy	58 (64.4)
Breast conserving surgery	32 (35.6)
Dose regimen, *n* (%)	
Conventional	63 (70.0)
Hypofractionated	27 (30.0)
Tumor bed boost PTV, *n* (%)	34 (37.8)
Supraclavicular fossa irradiation, *n* (%)	73 (81.1)
Axilla irradiation, *n* (%)	20 (22.2)
Internal mammary chain (IMC) irradiation, *n* (%)	29 (32.2)
Radiation technique, *n* (%)	
Tangent	81 (90.0)
VMAT	9 (10.0)
Age (years), median (IQR)	44.0 [36.0–46.0]
BMI (kg/m^2^), mean ± SD	29.52 ± 5.15

Abbreviations: PTV, planning target volume; IMC, internal mammary chain; VMAT, volumetric modulated arc therapy; and BMI, body mass index.

**Table 2:** Comparison of lung and heart doses between free breathing (FB) and deep inspiration breath hold (DIBH).

Dosimetric Parameter	FB Mean ± SD	DIBH Mean ± SD	Mean Difference ± SD	95% CI of Difference	Cohen’s d	*p*-Value
Mean Lung Dose	1400.08 ± 345.38	1163.70 ± 331.87	236.38 ± 86.49	218.26–254.49	2.73	<0.001
Mean Heart Dose	424.73 ± 147.04	264.25 ± 128.42	160.48 ± 65.12	146.84–174.12	2.46	<0.001

FB = free breathing; DIBH = deep inspiration breath hold; and Cohen’s d calculated from SD of paired differences. Mean difference = FB − DIBH.

**Table 3:** Comparison of heart V5 and lung V20 between FB and DIBH.

Dosimetric Parameter	FB Median [IQR]	DIBH Median [IQR]	Median Difference [IQR]	Effect Size (r)	*p*-Value
Heart Dose V5	0.165 [0.05–0.398]	0.060 [0.00–0.214]	0.105 [0.03–0.214]	0.87	<0.001
Lung Dose V20	0.280 [0.10–0.50]	0.225 [0.05–0.36]	0.055 [0.03–0.09]	0.87	<0.001

Effect size r = Z/√*N*; *p*-values from the Wilcoxon signed-rank test. Median difference = FB − DIBH.

**Table 4:** Univariable correlation between continuous patient- and treatment-related predictors and dosimetric reductions (ΔMHD, ΔMLD, ΔLung V20, and ΔHeart V5).

Predictor	Δ%MHD (r, *p*)—Pearson	Δ%MLD (r, *p*)—Spearman	Δ%Lung V20 (r, *p*)—Spearman	Δ%Heart V5 (r, *p*)—Spearman
Age	−0.015 (0.886)	0.052 (0.629)	−0.073 (0.493)	0.152 (0.152)
BMI	−0.436 (<0.001)	−0.217 (0.040)	−0.255 (0.015)	−0.093 (0.383)
Threshold	0.335 (0.001)	0.198 (0.061)	0.392 (<0.001)	0.229 (0.030)

Δ%: relative reduction. Pearson correlation was used for normally distributed outcomes; Spearman correlation was used for non-normally distributed outcomes.

**Table 5:** Univariable analysis of binary patient- and treatment-related predictors associated with dosimetric reductions (ΔMHD, ΔMLD, ΔLung V20, and ΔHeart V5).

Predictor	Group Comparison	Δ%MHD Mean Diff [95% CI]	*p*-Value	Cohen’s d	Δ%MLD Mean Rank	*p*-Value	r	Δ%Lung V20 Mean Rank	*p*-Value	r	Δ%Heart V5 Mean Rank	*p*-Value	r
Surgical procedure	Mastectomy vs. Breast Conserving Surgery	2.8 [−3.6, 9.3]	0.381	0.194	44.16 vs. 47.94	0.511	−0.069	43.15 vs. 49.77	0.250	−0.121	45.21 vs. 46.03	0.886	−0.015
Dose regimen	Conv. vs. Hypo.	−9.1 [−15.6, −2.7]	0.006	−0.646	38.90 vs. 60.89	<0.001	−0.386	39.84 vs. 58.70	0.002	−0.331	39.99 vs. 58.35	0.002	−0.322
Tumor bed boost	No vs. Yes	3.9 [−2.4, 10.3]	0.218	0.270	44.77 vs. 46.71	0.733	−0.036	43.88 vs. 48.16	0.451	−0.079	46.44 vs. 43.96	0.662	−0.046
SCF	No vs. Yes	3.7 [−4.1, 11.6]	0.349	0.254	68.12 vs. 40.23	<0.001	−0.417	63.06 vs. 41.41	0.002	−0.325	52.06 vs. 43.97	0.250	−0.121
Axilla	No vs. Yes	4.6 [−2.7, 12]	0.217	0.316	46.89 vs. 40.65	0.346	−0.099	45.64 vs. 45	0.923	−0.010	47.61 vs. 38.10	0.151	−0.151
IMC	No vs. Yes	5.9 [−0.58, 12.4]	0.074	0.408	51.10 vs. 33.72	0.003	−0.311	48.76 vs. 38.64	0.086	−0.181	50.11 vs. 35.81	0.015	−0.256
Technique	Tangent vs. VMAT	6.0 [−4.2, 16.2]	0.245	0.411	45.73 vs. 43.44	0.804	−0.026	45.30 vs. 47.28	0.830	−0.023	47.27 vs. 28.67	0.041	−0.215

ΔMHD: percent reduction in mean heart dose; ΔMLD: percent reduction in mean lung dose; Δlung V20: percent lung volume receiving ≥20 Gy; and Δheart V5: percent heart volume receiving ≥5 Gy. Conv.: conventional fractionation; Hypo.: hypofractionation; SCF: supraclavicular fossa; and IMC: internal mammary chain.

**Table 6:** Multivariable analysis of clinical factors associated with cardiac and pulmonary dosimetric reductions with DIBH.

Predictor	Δ%MHD B (*p*-Value)	95%CI	Δ%MLD B (*p*-Value)	95%CI	Δ%Lung V20 B (*p*-Value)	95%CI	Δ%Heart V5 B (*p*-Value)	95%CI
BMI	−1.398 (<0.001)	−1.923, −0.873	−0.313 (0.019)	−0.573, −0.053	−0.365 (0.086)	−0.783, 0.053	−0.77 (0.082)	−1.64, 0.101
Dose Regimen (Conv vs. Hypo)	8.882 (0.007)	2.472, 15.292	3.723 (0.035)	0.262, 7.183	5.466 (0.054)	−0.102, 11.033	12.355 (0.023)	1.729, 22.981
SCF	-	-	−4.675 (0.017)	−8.486, −0.864	−8.81 (0.005)	−14.948, −2.685	-	-
IMC	−3.450 (0.276)	−9.708, 2.809	−1.559 (0.324)	−4.684, 1.566	−0.435 (0.864)	−5.463, 4.593	−8.29 (0.116)	−18.674, 2.077
Age	0.1 (0.457)	−0.166, 0.37	0.042 (0.531)	−0.090, 0.173	−0.023 (0.832)	−0.234, 0.189	0.305 (0.173)	−0.137, 0.747

Conv.: conventional fractionation (reference category); Hypo.: hypofractionation; SCF: supraclavicular fossa; and IMC: internal mammary chain. Positive B values indicate greater dosimetric reduction (more benefit) with DIBH, while negative values indicate smaller reductions (less benefit).

## Data Availability

The data that support the findings of this study are available from the corresponding author upon reasonable request. The data are not publicly available due to patient privacy concerns and institutional ethical restrictions.

## Supplementary Materials

The following supporting information can be downloaded at: https://www.mdpi.com/article/10.3390/diagnostics16081236/s1, Table S1. Comparison of delta_MHD and delta_Lung_Mean between smoking groups. Table S2. Kruskal–Wallis Test for ΔLung_V20 and ΔHeart_V5 According to Smoking Status.

## Abbreviations

ABC—Active Breath Control; BMI—Body Mass Index; CT—Computed Tomography; DIBH—Deep Inspiration Breath Hold; FB—Free Breathing; Gy—Gray; IHD—Ischemic Heart Disease; IMC—Internal Mammary Chain; LAD—Left Anterior Descending (artery); MHD—Mean Heart Dose; MLD—Mean Lung Dose; OAR—Organ at Risk; RNI—Regional Nodal Irradiation; RT—Radiation Therapy; SCF—Supraclavicular Fossa; and VMAT—Volumetric Modulated Arc Therapy.
