# Supplementary material for: Clinical Predictors of Dosimetric Precedence for Deep Inspiratory Breath Hold Radiation Therapy for Breast Cancer
Source: Diagnostics (Basel). 2026 Apr 21;16(8):1236. doi: 10.3390/diagnostics16081236 (PMC13115145; doi:10.3390/diagnostics16081236)
Supplement: Supplementary file 1 [file diagnostics-16-01236-s001.zip › Supplementary Tables-DIBH.pdf]

Clinical predictors of dosimetric precedence for deep inspiratory breath-hold radiation therapy for breast cancer

Table S1 Comparison of delta_MHD and delta_Lung_Mean between smoking groups

**Variable**	**Smoking Status**	**N**	**Mean ± SD**	**95% CI for Mean**	**Min–Max**	**ANOVA *p*-value**
**ΔMHD**	No	66	161.2 ± 65.6	145.0 – 177.3	34.5–298.8	0.986
	Yes	18	158.4 ± 71.0	123.1 – 193.7	27.0–278.0	
	Ex-smoker	6	159.4 ± 49.2	107.7 – 211.0	72.0–218.4	
**ΔMLD**	No	66	235.2 ± 92.3	212.5 – 257.8	51.0–452.0	0.545
	Yes	18	228.7 ± 67.5	195.1 – 262.2	150.0–364.0	
	Ex-smoker	6	273.0 ± 71.2	198.3 – 347.7	142.0–343.0	

Table S2. Kruskal–Wallis Test for ΔLung_V20 and ΔHeart_V5 According to Smoking Status

**Outcome**	**Smoking Status**	**N**	**Mean Rank**	**χ^2 (df)**	**p-value**
**Δ Lung V20**	No	66	44.18		
	Yes	18	50.89	0.973 (2)	0.615
	Ex-Smoker	6	43.83		
**Δ Heart V5**	No	66	43.33		
	Yes	18	49.64	2.084 (2)	0.353
	Ex-Smoker	6	57		
